# Multifaceted Roles of Caveolin-1 in Lung Cancer: A New Investigation Focused on Tumor Occurrence, Development and Therapy

**DOI:** 10.3390/cancers12020291

**Published:** 2020-01-25

**Authors:** Yu-Bo Shi, Jun Li, Xing-Ning Lai, Rui Jiang, Rui-Chen Zhao, Li-Xia Xiong

**Affiliations:** 1 Department of Pathophysiology, Basic Medical College, Nanchang University, Nanchang 330006, China; 15797899116@163.com (Y.-B.S.); lj012729@163.com (J.L.); laixingning99@outlook.com (X.-N.L.); jp6300517180@qmul.ac.uk (R.-C.Z.); 2 Queen Mary School, Jiangxi Medical College of Nanchang University, Nanchang 330006, China; jp6303416077@qmul.ac.uk; 3 Second Clinical Medical College, Nanchang University, Nanchang 330006, China; 4 Jiangxi Province Key Laboratory of Tumor Pathogenesis and Molecular Pathology, Nanchang 330006, China

**Keywords:** Caveolin-1 (Cav-1), lung cancer, tumor progression, metastasis, targeted therapy

## Abstract

Lung cancer is one of the most common and malignant cancers with extremely high morbidity and mortality in both males and females. Although traditional lung cancer treatments are fast progressing, there are still limitations. Caveolin-1 (Cav-1), a main component of caveolae, participates in multiple cellular events such as immune responses, endocytosis, membrane trafficking, cellular signaling and cancer progression. It has been found tightly associated with lung cancer cell proliferation, migration, apoptosis resistance and drug resistance. In addition to this, multiple bioactive molecules have been confirmed to target Cav-1 to carry on their anti-tumor functions in lung cancers. Cav-1 can also be a predictor for lung cancer patients’ prognosis. In this review, we have summarized the valuable research on Cav-1 and lung cancer in recent years and discussed the multifaceted roles of Cav-1 on lung cancer occurrence, development and therapy, hoping to provide new insights into lung cancer treatment.

## 1. Introduction

Globally, lung cancer occupies the highest incidence and mortality among all carcinomas in males. In females, the incidence of lung cancer is the third highest after breast cancer and colorectum cancer, while its mortality is only secondary to breast cancer [1]. Besides the original disease, lung cancer patients also tend to have comorbidities, such as chronic obstructive pulmonary disease, cardiovascular diseases, cerebrovascular disorders and diabetes, which further threaten their lives [2]. In a clinical scenario, lung carcinoma can be classified into small cell lung cancer (SCLC) and non-small cell lung cancer (NSCLC) based on cell size. They have significant differences in cellular morphology, malignancy and prognosis. SCLC accounts for approximately 15% of all the patients. The other 85% are NSCLC cases. Compared with NSCLC, SCLC is more aggressive and malignant, with a low 5-year survival rate of 5%. NSCLC is a large group containing squamous cell carcinoma (SCC), adenocarcinoma (AC) and large cell lung cancer (LCLC), and it has a good prognosis through surgical interference at the early stage [3].

**Figure 1:**
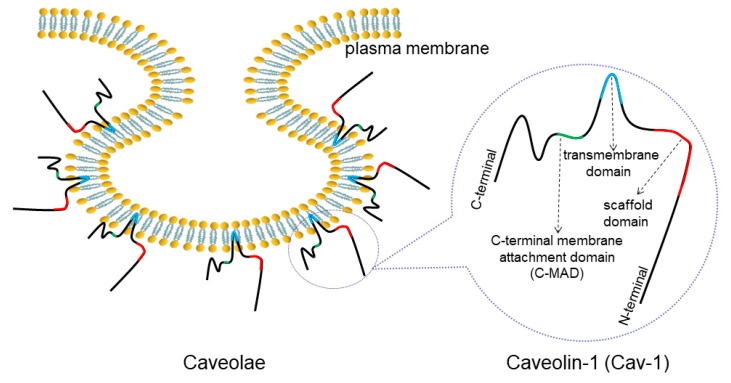
Structure of caveolae and Cav-1. Caveolae is an Ω-shaped structure located on cell membranes. Cav-1 is a main component of caveolae, consisting of a C-MAD, transmembrane, and scaffolding domains.

Caveolin-1 (Cav-1) is one of the main constituents of Ω-shaped caveolae on the plasma membrane (Figure 1). The abnormal expression of Cav-1 detected in lung cancer has been closely linked to cancer progression such as cell proliferation, migration, apoptosis and drug resistance. Therefore, it is necessary to decipher the detailed correlation between Cav-1 and lung cancer.

## 2. Overview of Caveolae and Cav-1

Caveolae are described as flask-shaped, invaginated structures on the plasma membrane that can also fuse to form grape-like structure and tubules [4]. Caveolae are predominantly located in stressed cells like muscle cells, endothelial cells, fibroblasts and adipose tissues, but are undetectable in neurons, lymphocytes and kidneys [5,6]. Caveolae have basic roles including membrane trafficking, lipid metabolism and cell signal transduction, and are associated with various diseases [7,8]. Studies have revealed that the main components of caveolae, the caveolins (Cavs) family, contain three isoforms: Cav-1, Cav-2 and Cav-3. Besides the specificity of caveolae distribution, the Cavs expression also presents tissue-specific variation. It has been proven that both Cav-1 and Cav-2 are expressed in all tissues excluding skeletal muscle, while Cav-3 is mainly expressed in striated muscle. Some particular cells or tissues, for instance, the cardiac muscle and smooth muscle, can simultaneously express Cav-1, Cav-2 and Cav-3 [7,8,9,10]. The Cavin family is another group of key molecules to form the caveolae. The Cavin family includes cavin-1 (polymerase I and transcript release factor, PTRF), cavin-2 (serum deprivation response protein, SDPR), cavin-3 (serum deprivation response factor-related gene product that binds to C-kinase, SRBC) and cavin-4 (muscle-restricted coiled-coil, MURC) [11]. Cavins can remodel the plasma membrane by interacting with membrane lipids, and regulate caveolae assembly [12]. Cavin-1 can prevent the degradation of Cav-1 from lysosomal enzymes through interacting with Cav-1 to shape caveolae structure [13]. The complex is fixed firmly to the membrane, which is essential for caveolae formation [13,14].

Cav-1 is identified as one of the main components of caveolae with 22kDa molecular weight, and the *Cav-1* gene is located at the D7S522 locus on chromosome 7 (7q31.1) with three exons [15]. Cav-1 plays multiple roles in immune responses, endocytosis, membrane trafficking, cellular signaling, and is also related to specific diseases such as atherosclerosis, pulmonary hypertension and Alzheimer’s disease [8,9]. Particularly, Cav-1 is found to be associated with cell differentiation, proliferation, migration and invasion in cancers [16]. The roles of Cav-1 in cancers are controversial. In some cancers, such as colorectal cancer [17] and ovarian cancer [18], Cav-1 expression is down-regulated, suggesting that Cav-1 can inhibit such cancer development. Interestingly, it is elevated in other malignancies such as endometrial carcinoma [19], hepatic cancer [20], breast cancer [21], prostate cancer [21], and pancreatic cancer [22], in which Cav-1 propels cell growth and migration and results in cancer deterioration. This dual role has been found to be stage-dependent, since Cav-1 is downregulated and performs tumor-suppressor function at the early stage, while at the later stage, Cav-1 is up-regulated and plays oncogenic roles [16].

The context-dependent role of Cav-1 is seen also in lung cancer. Cav-1 expression is greatly reduced in lung cancer compared with the normal pulmonary tissue, and its expression in cancer tissues with different histological types and stages also shows variation (Table 1). The expression of Cav-1 in parenchyma is higher in SCLC than in NSCLC, and is lower at the advanced stage than at the early stage. Even in the same tissue, its expression in individual cells can be distinct from each other, shown by immunohistochemistry (IHC) staining. Furthermore, it can also be totally absent in some other cases [23,24,25]. In lung cancer, Cav-1 is found to act on multiple downstream effectors, such as epidermal growth factor receptor (EGFR) [26], extracellular regulated protein kinases (ERK) [27], focal adhesion kinase (FAK) [28] and protein kinase B (AKT) [28], to mediate key aspects of cancer progression. Due to these functions, Cav-1 can be considered to act as a target for lung cancer therapy.

## 3. Cav-1-Mediated Lung CSC Generation

Cancer stem cells (CSC) are a subset of cells with self-renewal capacity and tumorigenic potential within a tumor. It can consequently contribute to tumorigenesis, growth, migration, drug resistance and recurrence in cancers. CSCs are characterized by their spheroidal shape, the expression of specific cell markers, and their ability to pump out anti-tumor agents through membrane transporters, leading to drug resistance [36,37,38]. The transcription factor octamer-binding transcription factor 4 (Oct-4) is reported to play an important role in the stem cell generation during embryogenesis and tumorigenesis by regulating the targeted genes transcription [39,40]. In H460 NSCLC cells, Cav-1 can bind Oct-4 to enhance its degradation via the ubiquitin-proteasome pathway. Nitric oxide (NO) treatment of H460 cells can disrupt the complex and free Oct-4 from Cav-1, thus allowing Oct-4 to exert its action in the nucleus and bringing CSC characteristics to this cell line [41]. The antibiotic ciprofloxacin is found to upregulate Cav-1 in H460 cells and subsequently to impel activated AKT and ERK expressions. Both of them jointly induce the increase in Oct-4 expression, which eventually converts the original cancer cells to CSC-like cells [42]. In addition, culturing human lung epithelial BEAS-2B cells with single-walled carbon nanotubes (SWCNTs) will confer such cells with CSC characteristics. During this process, SWCNT exposure can increase Cav-1 levels, which can inhibit the downstream tumor suppressor molecule p53 to promote CSC induction and lung cancer progression [43,44]. The latter findings strongly suggest a positive correlation between Cav-1 and lung CSCs generation.

## 4. Cav-1 Regulates Lung Cancer Cell Proliferation

Cell proliferation is a cycle that consists of G1, S, G2 and M phases, which can usually be regulated by cyclin (cyclin A, B, D and E) and cyclin-dependent kinase (CDK; CDK1, 2, 4 and 6) protein families. Abnormal expression of these regulators can lead to uncontrolled cell proliferation, and then tumor development [45,46,47].

Cav-1 is involved in the lung cancer cell cycle. Typically, in a majority of NSCLC cell lines (H460 and A549), Cav-1 has oncogenic characteristics that facilitate lung cancer cell proliferation. In H460 cells, Cav-1 knockdown can reduce the expression of cyclin D1 and proliferating cell nuclear antigen (PCNA, a regulator of DNA replication during cell division). Subsequently, most cells pause at the G1/S stage, thus inhibiting cell proliferation [48]. In paclitaxel-resistant A549 cells, Cav-1 knockdown results in decreased proliferation by reducing cyclin D1 expression and inhibiting AKT phosphorylation. As a result, the dividing cells are arrested at the G0/G1 phase and the number of cells in the S or G2/M phase is dramatically reduced [49]. While in SCLC cell lines (SCLC-R1, H446), Cav-1 can play dual roles on regulating cell proliferation. The proliferation inhibition is found in Cav-1-knockdown SCLC-R1 and RAL cells. Due to Cav-1 downregulation, the cyclin D1 and CDK4 levels are both reduced. Meanwhile, the expression and phosphorylation of the signal transducer and activator of transcription 3 (STAT3), a key transcription factor targeting gene *CCND1* (can encode cyclin D1), are also decreased. Reduced expression of cyclin D1 can eventually lead to slow cell division. These factors contribute to cell growth arrest all together [50]. Such cases indicate that Cav-1 knockdown can inhibit lung cancer cell proliferation via negatively regulating the cell cycle, which suggests a probably positive correlation between Cav-1 and lung cancer cell proliferation. However, Sun et al. drew a completely opposite conclusion in H446 cells. They found that Cav-1 over-expression could decrease pERK1/2 expression and make most cells arrest at the G2/M phase, and finally inhibit cell proliferation [27]. In the study, they also found that Cav-1 over-expression could lead to estrogen receptor (ER) and progesterone receptor (PR) reductions. Estrogen and progesterone have been reported to stimulate cell proliferation in breast cancer by elevating cyclin G1 expression [51]. However, the direct evidence of Cav-1-mediated cell proliferation by acting on ER and PR still lacks. This is probably the reason why this cell line behaves contrary to the others. Cav-1 can also facilitate lung cancer cell proliferation via other pathways. In A549 and GLC-82 cells, Cav-1 can function as one of the plasma membrane components to mediate EGFR endocytosis with the help of prostaglandin E2 (PGE2), to induce its nuclear translocation. Then EGFR can interact with STAT3 in the nucleus and promote STAT3 activation, leading to enhanced cell proliferation. Thus, Cav-1 ablation will impair EGFR accumulation within the nucleus and restrain cell proliferation [52]. Cav-1 over-expression can increase GLC-82 cell proliferation, and an obvious tumor growth was also observed in mice models transplanted with such cells [26]. In A549 and SK-MES-1 cells, by RT-qPCR, lncRNA HOTAIR expression was checked and shown to be increased by Cav-1. Then, as was shown in the results of CCK-8 and transwell assays, they can synergistically facilitate cell proliferation and viability [53].

## 5. Cav-1 Participates in Lung Cancer Metastasis

**Figure 2:**
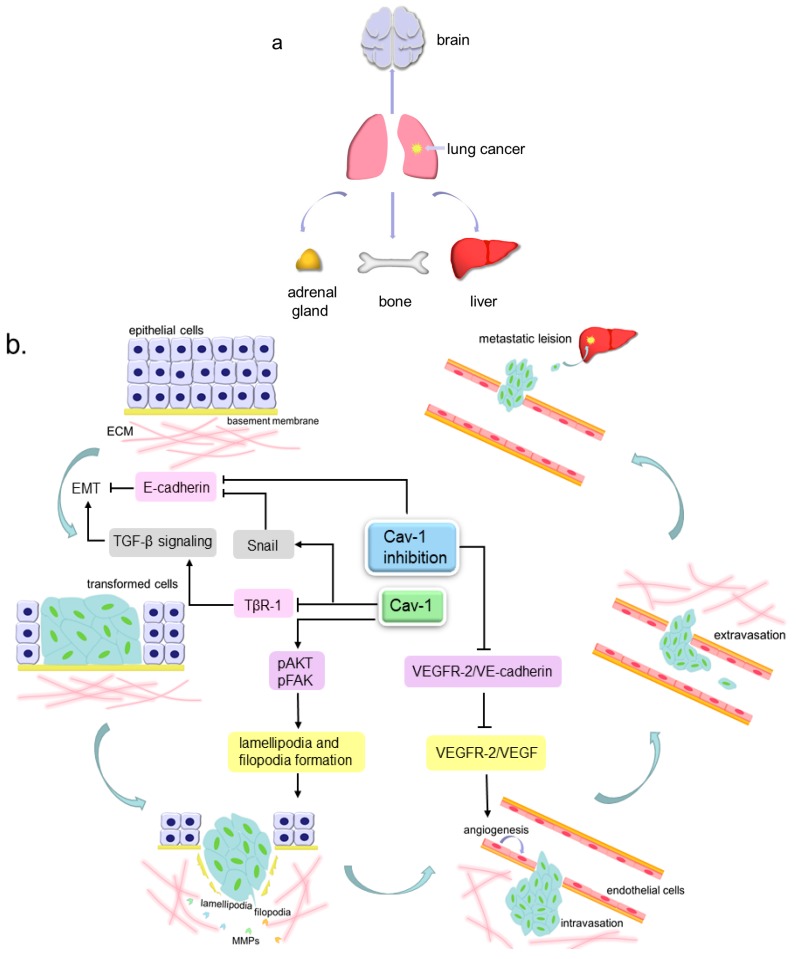
The pattern of lung cancer metastasis. (**a**). The preferential sites of lung cancer metastasis, include brain, liver, bones and adrenal glands; (**b**). Cav-1 participates in lung cancer metastasis. The lung tumor epithelial cells can undergo epithelial to mesenchymal transition (EMT) to become more invasive transformed cells, then invade locally into the stroma by extending motive pseudopods and secreting MMPs to degrade ECM, later intravasate into blood or lymphatic vessels and survive within the circulation, and finally extravasate and generate a metastatic lesion at a distant site. Cav-1 can inhibit TGF-β signaling-mediated EMT via inducing the internalization and degradation of TβR-1. Cav-1 over-expression can increase the Snail level to repress the E-cadherin level, further to facilitate EMT, and can also increase the lamellipodia and filopodia formation by elevating pAKT and pFAK expression, leading to enhanced cell invasiveness. Cav-1 absence can prevent VEGFR-2 from binding VE-cadherin and permits VEGFR-2 to be phosphorylated by VEGF, thus to enhance endothelial cell growth and lead to angiogenesis. Cav-1 knockdown can reduce E-cadherin to attenuate cell adhesion and lead to EMT.

Cell migration is a process that can take place in both physiological and pathological conditions, such as early embryo development, tissue repair, immune response, inflammatory response and cancer metastasis [54]. Metastasis consists of a series of events that can be described as follows: cancer cells detach from extracellular matrix (ECM), then invade locally into the stroma by secreting matrix metalloproteinases (MMPs) to degrade ECM, later intravasate into blood or lymphatic vessels and survive within the circulation, and finally the cells extravasate and generate a metastatic lesion at a distant site (Figure 2) [55,56]. There are three different routes for metastasis, namely, direct seeding, the hematogenous pathway and lymphocytic pathway. Lung cancer undergoes metastasis through either blood vessels or lymphatic vessels in most cases. The most common sites that the secondary lung cancer preferentially occurs are bones, liver, brain and adrenal glands [57]. A clinicopathological test has unveiled that Cav-1-positive lung AC patients tended to have worse tumor, node, and metastasis (TNM) stage in comparison to the negative patients [30]. Hence, Cav-1 may play a significant role in lung cancer cell migration.

### 5.1. Cav-1 and Angiogenesis

Like other cancers, lung cancer is composed of parenchyma and stroma. Parenchyma is the real tumor mass that consists of true neoplastic cells. The major components of stroma are connective tissues and blood vessels. They can support and nourish the parenchymal portion. To meet the nutritional requirements, new blood vessels in lung cancer are constantly formed based on the existing networks in the stroma, which can also provide pathways for cancer cell hematogenous migration [58,59,60]. In addition, the vasculature in cancers is more permeable than that in normal tissues, therefore making it easier for cancer cells to migrate [61]. Vascular endothelial growth factor (VEGF) family proteins can be secreted by tumor cells. They can bind to the VEGF receptor (VEGFR) on the endothelial cell membrane to regulate endothelial cell proliferation and migration, which is regarded as angiogenesis [62,63,64].

Some studies have demonstrated the links between Cav-1 and the angiogenesis that occurs in lung cancer. In Lewis lung carcinoma (LLC) cells (NSCLC cell line), cavtratin, the scaffolding domain peptide of Cav-1, can inhibit endothelial nitric oxide synthase (eNOS) function and subsequently block NO production, eventually to prevent LLC cells leakage and decrease angiogenesis [61,65,66,67]. In Cav-1-knockout mice transplanted with LLC cells, the Cav-1 absence prevents VEGFR-2 from binding VE-cadherin (a protein that can interact with VEGFR-2 and lead to its dephosphorylation) and permits VEGFR-2 to be phosphorylated by other factors such as VEGF families, thus to enhance endothelial cell growth and lead to angiogenesis. Such mice also show microvascular hyperpermeability [68,69]. These findings imply that Cav-1 can negatively regulate lung cancer angiogenesis and prevent tumor cell leakage.

### 5.2. Cav-1 and Pseudopods Formation

Pseudopods, including filopodia, lamellipodia and invadopodia, are cellular protrusions that can mediate cell motility and sensation [54]. Filopodia and lamellipodia have similar functions but are structurally different. Filopodia are finger-like protrusions while lamellipodia are flatter and broader, both of which are important for cell migration [70]. There are complex and massive molecules associated with pseudopods formation. FAK is a non-receptor tyrosine kinase that is up-regulated in various cancers, and involved in tumor cell proliferation, migration and survival. By recruiting the downstream molecules paxillin and talin, FAK is able to modulate focal adhesion dynamics to control cell motility [71,72]. It can also interact with the p85 subunit of phosphatidylinositol 3-kinase (PI3K) to phosphorylate AKT [73,74]. AKT can mediate actin organization by acting on Girdin, an actin-binding protein [75,76]. Actin polymerization and rearrangement eventually trigger the generation of pseudopods [77,78].

Cav-1 can mainly induce the formation of filopodia and lamellipodia, thus enhancing NSCLC cell migration. In NO-treated H460 cells, Cav-1 expression is upregulated, which subsequently elevates phosphorylated FAK (pFAK) and phosphorylated AKT (pAKT) levels. As a result, such cells generate more filopodia and show stronger motility and invasiveness [28]. In H23 cells, with a fluorescence microscope, the lamellipodia number is observed to be increased due to Cav-1 over-expression, which can elevate pAKT expression leading to enhanced cell invasiveness [79]. Similar to the aforementioned results, Ho et al. also reported that in the highly-invasive lung AC cell lines (CL1-5 and CL1-5F4), Cav-1 expressed higher than that in the low-invasive cell lines (CL1-0 and CL1-1). Such high expression of Cav-1 induced the formation of filopodia and eventually enhanced cell motility. Additionally, they demonstrated that the lipid existence was indispensable for Cav-1-mediated filopodia formation [80]. On the contrary, in H460 and H292 cells, chrysotobibenzyl-induced Cav-1 downregulation leads to decreases in pFAK and pAKT, which suppresses filopodia generation and cell migration [81].

### 5.3. Cav-1 and EMT 

During EMT, cells lose their original characteristics and gain mesenchymal properties. The original cobblestone-like epithelial cells are converted to spindle-like mesenchymal cells. The expression of epithelial markers, such as E-cadherin, are downregulated, while mesenchymal markers such as N-cadherin, Vimentin, Snail, Slug, Twist and alpha-smooth muscle actin (α-SMA) are over-expressed. Such cells can also secrete MMPs to degrade the basement membrane, thus permitting the cells to invade and migrate [82,83,84,85].

In NSCLC cell lines, Cav-1 can exert dual roles on EMT progression. In A549 cells, transforming growth factor-β (TGF-β) can bind to the TGF-β receptor-1 (TβR-1) on the cell membrane, which triggers EMT by upregulating Vimentin, Snail and Slug, and downregulating E-cadherin. Cav-1 can also combine with TβR-1 to induce its internalization and further cause its degradation mediated by ubiquitin-proteosome, thus blocking TGF-β signaling pathway. In contrast, syntenin, a scaffold protein that can interact with multiple membrane receptors, is able to prevent TβR-1 internalization and degradation by disassociating the TβR-1/Cav-1 complex, finally to facilitate lung cancer progression [86,87]. A metastasis-associated protein called EF-hand domain-containing protein D2 (EFHD2) is found to inhibit Cav-1 mRNA and protein expressions, and subsequently upregulate Snail and Vimentin as well as downregulate E-cadherin in A549 cells to enhance EMT. Additionally, Cav-1 re-expression can partly reverse EFHD2-induced EMT [88]. E-cadherin can link to β-catenin on the cell membrane and then strengthen cell adhesion. In H460 cells, Cav-1 knockdown can reduce E-cadherin and increase β-catenin, hence, attenuating cell adhesion leading to EMT process [48,89]. The aforementioned studies suggest an inhibitory role of Cav-1 in regulating EMT in NSCLC. However, in some occasions, Cav-1 may function as a positive regulator. In H157 cells, Cav-1 over-expression can increase the Snail level, which further represses the E-cadherin level to facilitate EMT and migration [90,91]. Conversely, in Cav-1-knockdown H460 and 95D cells, decreased pEGFR, pERK1/2, MMP-2 and MMP-9 levels can be measured by western blot and reduced cell invasion can be observed by transwell assay [92,93]. In Cav-1 over-expressed SCLC cell line H446, EMT-associated characteristics such as low E-cadherin expression and high MMP-3 expression can be detected, resulting in enhanced cell invasion [94].

### 5.4. Cav-1 Regulates Cell Movement via Multiple Molecules

There are some other pathways involved in Cav-1-mediated NSCLC cell motility. Located on the cell membrane, caveolae can sense the alteration of mechanical stress, and Cav-1 can be phosphorylated responding to several stimuli, thus, to be involved in multiple cellular events [6]. Sinha et al. found that mechanical stress over Hela and mouse lung endothelial cells flattened caveolae to buffer the tension and freed Cav-1 [95]. In A549 and CL1-5 cells, elevated hydrostatic pressure (which mimics increased interstitial fluid pressure that is seen in many solid cancers) can increase phosphorylated Cav-1. Then, through Cav-1/AKT/ERK axis, the aquaporin-1 (AQP1) level is elevated, which can impel cell migration [96]. In A549 cells, Cav-1 and protein arginine methyltransferase 5 (PRMT5) can synergistically facilitate the exteriorization of Eno-1, a glycolytic enzyme that can promote lung cancer cell migration. Eno-1 is further methylated by PRMT5 and then degrades the ECM for cell invasion [97]. Hydrogen peroxide (H_2_O_2_) and hydroxyl radical (·OH) are two key exogenous reactive oxygen species (ROS). In H460 cells, Cav-1 over-expression can elevate pAKT expression and subsequently suppress the production of H_2_O_2_ and ·OH. The dysregulation of ROS negatively influences the expression of vascular endothelial cell adhesion molecule-1 (VCAM-1). VCAM-1 can promote the adhesion between endothelium and cancer cells. Hence, its ablation makes such an attachment become loose, leading to promoted cell migration and invasion [98]. H_2_O_2_ and another ROS superoxide anion (O_2_^·−^) can decrease the Cav-1 level by enhancing its ubiquitination in H460 cells. Cav-1 downregulation then decreases pAKT, and results in attenuated cell migration and invasion. Nevertheless, ·OH treatment can produce entirely opposite results in comparison with the other two ROS [99]. Cav-1 can also function to inhibit lung cancer cell migration. In H1299 cells, Cav-1 can interact with DLC-1, an anti-metastasis protein, to co-localize in caveolae and form a complex. It can inhibit tumor cell migration probably by enhancing the tumor suppressive role of Cav-1 in these cells [100].

## 6. Roles of Cav-1 in Programmed Cell Death

Physiologically, cell proliferation and apoptosis are in a dynamic equilibrium. The dysregulation of such a balance can not only promote lung cancer cell survival but also accelerate its metastatic processes [101,102]. In lung cancer, the regulatory functions of Cav-1 on cell apoptosis are controversial. Some studies have demonstrated its inhibitory role in both SCLC and NSCLC cell lines. In H446 cells, a high Cav-1 level can stabilize B-cell lymphoma-2 (Bcl-2) expression and suppress caspase-3 activation, leading to decreased cell apoptosis induced by cisplatin and ultraviolet radiation treatments [103]. Conversely, the Cav-1 knockdown in A549 cells can repress the expressions of downstream molecules Rho-associated coiled-coil kinase 1 (ROCK1) and Parkin. Both of them are responsible for the repair of the injured mitochondria (regarded as mitophagy) in response to cisplatin treatment. Cav-1 silencing can also increase the production of ROS and the diffusion of cytochrome-c, therefore enhancing cell apoptosis [104]. In A549 and PC-9 cells, Cav-1 expression can be negatively modulated by its upstream regulator miR-204. The phosphorylation of AKT and Bad can be further suppressed by the miR-204/Cav-1 pathway, which can promote cisplatin-induced apoptosis by silencing Bcl-2 and Bcl-xl, indicating the positive role of Cav-1 during this process [105]. In paclitaxel-resistant A549 cells, Cav-1 knockdown upregulates Bax, caspase-3 and caspase-9, and downregulates Bcl-2, finally prompting cell apoptosis [49]. Converse studies have also shown that Cav-1 can induce lung cancer cell apoptosis in both histological types. In SHP77 cells, Cav-1 over-expression can reduce cadherin-11 expression to suppress STAT3 phosphorylation, further to induce an enhanced apoptotic rate, probably by regulating the transcription of genes controlling cell survival, including *Bcl-xL, Bcl-2* and *survivin* [106,107]. In H460 cells treated with cisplatin for 24 h, Pongjit et al. observed that Cav-1 could promote O_2_^·−^ production, and then enhance cisplatin-induced apoptosis [108]. As is mentioned above, Cav-1-overexpressed H446 cells show attenuated cell apoptosis after the treatment of cisplatin for 48 h [102]. In regard to the opposing conclusions, potential reasons could be that: (1) H460 and H446 are totally different cell lines (H446 is SCLC, H460 is NSCLC); (2) cisplatin may cause different results under differing treatment durations.

Anoikis is a programmed cell death induced upon cell detachment from the ECM behaving as a critical mechanism in preventing adherent-independent cell growth and attachment to an inappropriate matrix. It is an effective mechanism to prevent the scattered cells from sticking to a new type of tissue in a wrong place. Anoikis occurs both physiologically to maintain homeostasis and pathologically to regulate disease progressions [109,110,111]. If this mechanism is disrupted or deregulated, regarded as anoikis resistance, tumor cells will survive in the circulation and have metastasis potential [109]. Generally, Cav-1 can induce anoikis resistance in NSCLC cell lines. Being a member of the Bcl-2 family, myeloid cell leukemia-1 (Mcl-1) can inhibit apoptosis by suppressing the secretion of cytochrome c, which can activate caspases and give rise to cell death [112]. In H460 cells, the Cav-1 scaffold domain can interact with Mcl-1 to prevent its degradation induced by the ubiquitin-proteasomal pathway, and stabilize its expression, thus resulting in anoikis resistance [113]. Such effects can be negatively regulated by dendrofalconerol A (DF-A), which enhances the anoikis in H460 cells [114]. In H460 cells, H_2_O_2_ treatment can inhibit the ubiquitin-proteasomal pathway-mediated Cav-1 degradation to facilitate cell anoikis resistance [115]. Similarly, determined by immunoprecipitation, western blot and immunofluorescence, NO can trigger S-nitrosylation of Cav-1 and induce anoikis resistance in lung cancer [116].

## 7. The Relationship between Cav-1 and Lung Cancer Prognosis

Lung cancer prognosis can be mainly reflected by the 5-year survival rate of patients and re-occurrence incidence. Multiple physiological factors including age, gender, metabolism function, intensity of immune response and nutrition status can determine the lung cancer prognosis. In terms of biochemical prognostic factors, microRNA-197 (miR-197) [117], miR-145 [117] and programmed cell death-ligand 1 (PD-L1) [118] can be used to predict lung cancer outcomes.

Cav-1 is mainly a negative predictor for lung cancer outcomes. In lung SCC with lymph node metastasis, Cav-1 is mainly detected in stage III, and Cav-1-positive patients show a lower 5-year survival rate compared to the Cav-1-negative patients [33]. In lung AC, similarly, patients’ survival rate with high Cav-1 expression is greatly reduced [30]. It was also discovered in patients with LCLC and lung pleomorphic carcinoma that high Cav-1 expression contributed to a shorter disease-free and overall survival time [92,119]. Nevertheless, in some cases, Cav-1 can also act as a positive predictor. Duregon et al. identified that lung AC patients with proline-to-leucine mutations at codon 132 (P132L) of *Cav-1* had a longer survival time than patients without such mutations [120]. A study may partly account for this situation. In H1299 cells transfected with *Cav-1* P132L, such a mutant can interfere in the focal adhesion turnover to reduce cell migration and metastasis capacity, probably by attenuating filamin A phosphorylation [121]. NSCLC patients with a high level of stromal Cav-1 have a better partial response rate and overall survival in response to nab-Paclitaxel and carboplatin [122]. Instead, a low stromal Cav-1 level can predict a poor prognosis [123]. Interestingly, in some stroma tissue of primary lung tumors including AC, SCC and LCLC, Cav-1 and Cav-2 can be detected, however neither of them shows correlation with patients’ survival [124]. The possible reason for this may be that this investigation collected most of the cancer samples from early-stage patients, during which Cav-1 is weakly expressed or absent in most stromal cells, thus the correlation between Cav-1 expression and prognosis is not as significant as it was in other trials.

## 8. Cav-1 Involved in Lung Cancer Therapy

Current treatments of lung cancer include surgery, chemotherapy, radiotherapy and targeted therapy. The traditional surgical resection of the tumor is generally recommended for early-stage lung cancer (especially NSCLC) patients [125]. First-line drugs containing platinum (e.g., cisplatin and carboplatin) plus paclitaxel, and other chemotherapeutic agents such as gemcitabine, vinblastine and docetaxel are routinely applied for NSCLC chemotherapy [126,127]. Targeted drugs aimed at *EGFR* mutation, regarded as EGFR tyrosine kinase inhibitors (EGFR-TKI), are also well studied [128]. However, a major obstacle called multiple drug resistance (MDR) can usually affect drug efficacy when using these drugs as treatment.

### 8.1. Cav-1 and Drug Resistance

A high Cav-1 level has been shown to be associated with drug resistance during the treatment process of numerous cancers such as lung cancer, esophageal cancer [129], colorectal cancer [130] and renal carcinoma [131]. Cav-1 can enhance drug resistance in some NSCLC cell lines. In the parental paclitaxel-sensitive A549 cells, caveolae are absent. However, both caveolae and Cav-1 are upregulated in paclitaxel-resistant A549 cells, which implies a potential relationship between Cav-1 and paclitaxel resistance [132]. Conversely, Cav-1 knockdown in such paclitaxel-resistant A549 cells can induce apoptosis by increasing Bax and decreasing Bcl-2, thus, making the cells become sensitive to paclitaxel [49]. In PC-9 cells treated by gefitinib and erlotinib separately, Cav-1 knockdown results in attenuated cell proliferation and migration, and they become more sensitive to gefitinib and erlotinib. These are probably the consequences of Cav-1 downregulation-mediated decreases of pEGFR, pERK and pAKT. The same results were also observed in tumor-implanted nude mice treated exclusively with gefitinib [133]. In H292 cells, NO exposure can upregulate Cav-1, pAKT and Bcl-2, finally trigger chemo-resistance against doxorubicin and etoposide as well as promote cell survival, which were assessed by Hoechst 33342 staining and flow cytometry [134].

Unlike the aforementioned observations, some papers have also reported that Cav-1 can negatively regulate drug resistance in NSCLC cell lines. A high Cav-1 level could contribute to the entry of albumin and nab-paclitaxel (albumin-bound paclitaxel) into H23 lung cancer cells, therefore, enhancing cell apoptosis; while drug resistance is shown in these cells following Cav-1 reduction [135]. According to such a mechanism, a novel approach has been designed to maximize the efficacy of fenretinide. In A549 cells, Cav-1 upregulation can efficiently increase the absorption of fenretinide encapsulated by albumin shells, thus promoting fenretinide-induced toxicity in A549 cells [136]. P-glycoprotein (P-gp) can transport drugs from the cytosol to the matrix. Over-expression of P-gp can promote drug efflux, lower the intracellular drug concentration, reduce drug’s cytotoxicity, hence, leading to drug resistance [137,138]. It has been reported that P-gp is found highly expressed in caveolae-rich areas and can interact with Cav-1. Such an interaction can inhibit P-gp-mediated drug efflux to decrease drug resistance. For example, in doxorubicin-resistant Hs578T breast cancer cells, Cav-1 over-expression can reduce the activity of P-gp and corresponding doxorubicin resistance [139,140]. When the combination of Cav-1 with P-gp is interrupted, the function of P-gp can be reactivated. In A549 and H460 cells, Cav-1 with lysine 176-to-arginine (K176R) mutant fails to form the Cav-1/P-gp complex, thus P-gp functions normally to accelerate the efflux of substrates (i.e., paclitaxel and doxorubicin). Moreover, they also found that Cav-1 K176R mutant could influence Cav-1 oligomerization, which is essential for caveolae structure and is probably the cause for inducing the disassociation of Cav-1 and P-gp [141].

### 8.2. Cav-1 and Cell Senescence

Cell senescence refers to a physiologically inactive state and such cells undergo irreversible growth arrest [142]. Therefore, cell senescence can be regarded as a novel method of interrupting cancer cell proliferation and treating cancers [143]. In general, bleomycin can modulate Cav-1 expression, give rise to cell senescence and act as an anti-tumor agent [144]. In A549 cells, bleomycin treatment can elevate the Cav-1 level and then downstream molecules p53 and p21 [145]. Additionally, the Cav-1-induced activation of the p53/p21 pathway has been previously demonstrated to inhibit the function of CDK and inactivate DNA polymerase δ (a key regulator of DNA replication) to cause cell cycle arrest in NIH3T3 fibroblast cells [146]. Additionally, Cav-1 is also found to interact with MGr1-Ag on plasma membrane in the bleomycin-treated A549 cells. Their binding can potentially lead to cell senescence, however, further work is still required [147].

### 8.3. Multiple Cav-1-Targeted Agents

In the previous context, it has been discussed that multiple endogenous and exogenous agents can modulate Cav-1 expression to regulate lung cancer progression. There are also several natural bioactive compounds that can function similarly. They all target Cav-1 to inhibit NSCLC progression (Table 2). Some of them can inhibit lung cancer cell migration and invasion, such as chrysotobibenzyl and gigantol. Chrysotobibenzyl, a compound isolated from *Dendrobium pulchellum,* can suppress Cav-1 expression and then lead to the downregulation of integrin β1, β3, αv and downstream effectors pFAK and pAKT, thus inhibiting H292 and H460 cell migration [81]. In H460 cells, gigantol, a bibenzyl compound from *Dendrobium draconis,* can cause a decrease in Cav-1 levels and then pAKT to subsequently suppress Cdc42 expression and filopodia formation, finally to attenuate cell motility [148]. Others can elevate lung cancer cells' apoptotic rates, including dendrofalconerol A (DF-A), moscatilin, zinc, cordycepin, jorunnamycin A and ethanolic extract of *Antrodia cinnamomea* (EEAC). DF-A, a methanol extracted from *Dendrobium falconeri,* can elevate the sensitivity of H460 cells to anoikis by means of decreasing Cav-1, pAKT and Bcl-2 levels [113]. Moscatilin, isolated from *Dendrobium brymerianum,* can also induce anoikis by reducing pro-survival proteins pATK, pERK and Mcl-1 via a Cav-1-dependent pathway in H460 cells [149]. Zinc can sensitize H460 cells to anoikis by suppressing Cav-1 and pAKT [150]. In A549 cells, cordycepin, an adenosine analog, can raise Cav-1 expression. Cav-1 then elevates phosphorylated JNK (p-JNK) that can further prevent Foxo3a phosphorylation and enhance its nuclear translocation. In the nucleus, Foxo3a can increase the expression of Bax and caspase-3, then the cells exhibit reinforced apoptosis [151]. Jorunnamycin A is an extract from *Xestospongia sp.*, which can suppress Cav-1 expression to inhibit EMT and survival in H460, H292 and H23 cells [152]. By decreasing Cav-1 expression, EEAC can make A549 cells more sensitive to paclitaxel treatment and slow down cell viability [153].

## 9. Conclusions and Future Perspectives

**Figure 3:**
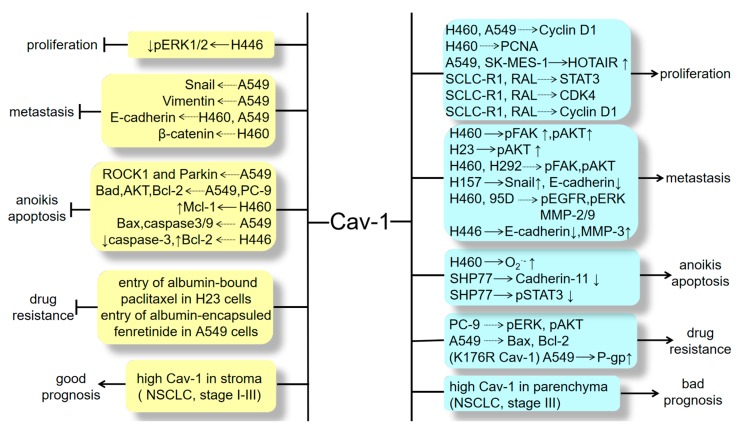
The dual roles of Cav-1 on regulating lung cancer development, including cell proliferation, metastasis, apoptosis, drug resistance and predicting patients’ survival. The dotted arrow indicates the mechanism that hasn't been elucidated clearly.

Currently, traditional therapies for the treatment of lung cancer still show some limitations during the treatment process. Distant metastasis and multi-drug resistance always badly influence therapeutic effects. Thus, it is necessary to identify novel molecular targets for effective targeted therapy. Cav-1 has been discovered to closely associate with lung cancer progression (Figure 3). In most cases, Cav-1 can positively regulate lung cancer development via promoting lung CSC transformation, cell proliferation, migration, drug resistance and decreasing cell apoptosis. Additionally, multiple biochemical compounds have been determined to target Cav-1 to exert various anti-NSCLC effects such as chrysotobibenzyl, gigantol, DF-A, moscatilin, cordycepin, Jorunnamycin A, EEAC, albumin-encapsuled fenretinide and bleomycin (Table 2). However, some studies also demonstrated its negative roles in different lung cancer stages and tissue types (Figure 3). As a result, its predictive function for lung cancer patients’ prognosis can be controversial. Although the complexity involving Cav-1-mediated lung cancer development has not been completely elucidated, it is also a potentially valuable target for treatment. Personalized medicine targeting Cav-1 against lung cancer could be a trend in the future as Cav-1 plays dual roles in different histological types, tumor stages and distributions (i.e., Cav-1 is distinctly expressed in tumor parenchyma and stroma), the therapeutic utilization of Cav-1 will be specific relying on patients’ individual conditions. Nowadays, studies on Cav-1 and lung cancer mostly focus on NSCLC progression, however, the research in SCLC is still lacking. Further studies are still required to explicitly identify the dual regulation of Cav-1 and the relevant molecular cascades in lung cancer.

## Figures and Tables

**Table 1:** The diversity of Cav-1 expression in non-cancer tissues and lung cancer tissues of different grades and types.

Tissue Type	Histological Type	Tumor Grade	Cav-1 positive Number/Total Sample Number	Reference
Non-cancer tissue	-----	-----	16/16(100%)	[29]
			15/19(78.9%)	[30]
			20/20(100%)	[31]
			20/20(100%)	[32]
Lung cancer tissue	AC	IV	23/116(19.8%)	[25]
	AC	I–III	19/43(44.19%)	[29]
	SCC	I–III	34/107(31.7%)	[33]
	AC+SCC+others (LCLC, ASC and carcinoid)	I–IV	60/115(52.2%)	[30]
	AC+SCC+LCLC	I–IV	105/160(65.7%)	[31]
	AC+SCC+LCLC	III and/or IV	12/73(16.4%)	[34]
	AC+SCC+LCLC+ASC	I–III	69/140(49.3%)	[32]
	SCLC	I–IV	49/70(70%)	[35]

ASC: adenosquamous carcinoma.

**Table 2:** Various Cav-1-targeted approaches to suppressing NSCLC progression.

Agent	Cell Line	Mode of Action	Reference
H_2_O_2_ and O_2_^·−^	H460	H_2_O_2_ and O_2_^·−^→ Cav-1↓→ pAKT↓→ migration↓	[99]
Chrysotobibenzyl	H460 H292	Chrysotobibenzyl→ Cav-1↓→ integrin β1, β3, αv↓ → pFAK↓ and pAKT↓→ migration↓	[81]
Gigantol	H460	Gigantol→ Cav-1↓→ pAKT↓→ CDC42↓→ EMT↓ and migration↓	[148]
DF-A	H460	DF-A→ Cav-1↓, Mcl-1↓and Bcl-2↓→ anoikis↑	[114]
Moscatilin	H460	Moscatilin→ Cav-1↓→ Mcl-1↓→ pAKT↓,pERK↓→ anoikis↑	[149]
Zinc	H460	Zinc→ Cav-1↓→ pAKT↓→ anoikis↑	[150]
Cordycepin	A549	Cordycepin→ Cav-1↑→ p-JNK↑→ Foxo3a↑→ Bax↑→ apoptosis↑	[151]
Jorunnamycin A	H460 H292 H23	Jorunnamycin A→ Cav-1↓→ pAKT↓,pERK↓→ EMT↓ and apoptosis↑	[152]
EEAC	A549	EEAC→ Cav-1↓→ chemosensitivity to paclitaxel↑	[153]
Albumin-encapsuled fenretinide	A549	Cav-1 promotes albumin-encapsuled fenretinide uptake into cell→ apoptosis↑	[136]
Bleomycin	A549	Bleomycin→ Cav-1↑→ p53↑, p21↑→ senescence↑	[145]
